# Supplementary figures and images for: The Hep-CORE policy score: A European hepatitis C national policy implementation ranking based on patient organization data
Source: PLoS One. 2020 Jul 28;15(7):e0235715. doi: 10.1371/journal.pone.0235715 (PMC7386634; doi:10.1371/journal.pone.0235715)

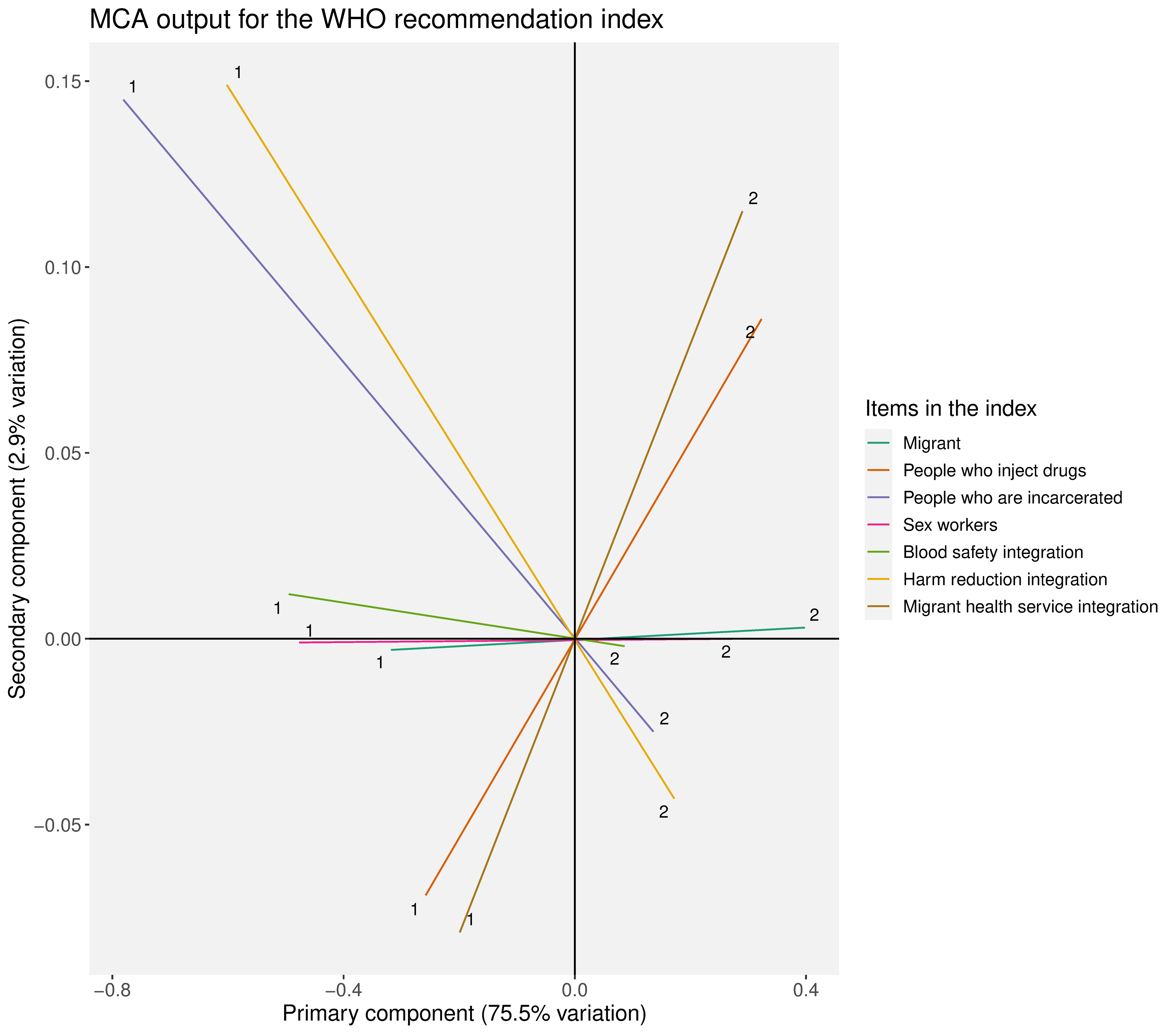

Supplement: S1 Fig — The MCA determined weight for all of the categories for the WHO recommendation index. (TIF) [file pone.0235715.s001.tif]

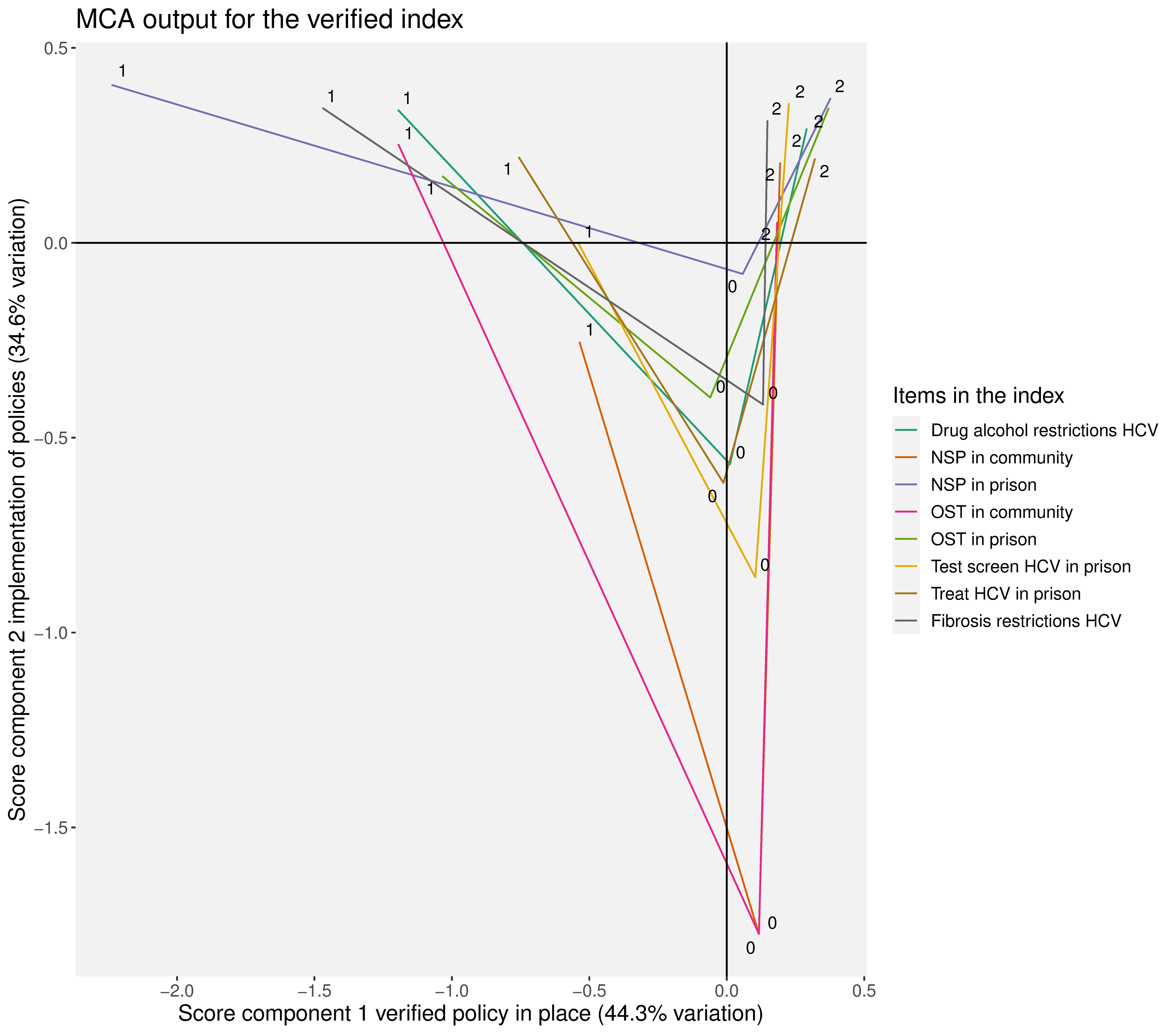

Supplement: S2 Fig — The MCA determined weights for all of the categories for the verified index. (TIF) [file pone.0235715.s002.tif]
