# Supplementary material for: The Hep-CORE policy score: A European hepatitis C national policy implementation ranking based on patient organization data
Source: PLoS One. 2020 Jul 28;15(7):e0235715. doi: 10.1371/journal.pone.0235715 (PMC7386634; doi:10.1371/journal.pone.0235715)
Supplement: S1 File — This is the complete survey that was used along with code fields. (PDF) [file pone.0235715.s003.pdf]

CHIP - Centre for Health & Infectious Disease Research

Hep-CORE 2018 Phase II

Codebook

Data Dictionary Codebook

2018-10-16 08:15:13

	#	Variable / Field Name	Field Label<br>*Field Note*	Field Attributes (Field Type, Validation, Choices, Calculations, etc.)
Instrument: **Hep-CORE 2018; Phase II** (hepcore_2018_phase_ii)				
	1	record_id	Record ID	text
	2	contact_info	Section Header: *Contact Information*<br>All personal and patient group contact information will remain private. Responses to the survey will be reported according to country and respective patient group.<br><br>Please provide the following information for the individual who will be most responsible for completing this survey.	descriptive
	3	contact_info_first	First name:	text, Required
	4	contact_info_last	Last name:	text, Required
	5	contact_info_org	Organisation:	text, Required
	6	contact_info_position	Position:	text, Required
	7	contact_info_street	Street address:	text, Required
	8	contact_info_postal	Postal code:	text, Required
	9	contact_info_city_2	City:	text, Required
	10	contact_info_country	Country:	dropdown (autocomplete), Required<br>1 Austria<br>2 Belgium<br>3 Bosnia & Herzegovina

4	Bulgaria
5	Croatia
6	Cyprus
7	Denmark
8	Egypt
9	Finland
10	France
11	Germany
12	Greece
13	Hungary
14	Israel
15	Italy
16	Macedonia
17	Netherlands
18	Norway
19	Poland
20	Portugal
21	Romania
22	Serbia
23	Slovakia
24	Slovenia
25	Spain
26	Sweden
27	Switzerland
28	Turkey
29	Ukraine
30	United Kingdom

	11	contact_info_phone	Phone number:<br>(Include country code, e.g. +45)	text, Required
	12	contact_info_email	Email address:	text (email), Required
	13	elim_efforts_hcv	Section Header: *Hep-CORE 2018 Phase II Questionnaire Section 1. Policies for Elimination*<br>Do you consider the status of current elimination efforts for hepatitis C in your country to be:<br><br>(see linked definition for WHO HCV elimination targets)	radio, Required<br>1 On track to reach WHO elimination targets by 2030<br>2 Working towards elimination<br>0 WHO elimination targets unachievable given present policy
	14	elim_efforts_hcv_ci_mx<br>Show the field ONLY if:<br>[elim_efforts_hcv] <>""	How informed do you consider yourself to be on the subject of this question?	radio (Matrix), Required<br>0 Not informed<br>3 Slightly informed<br>2 Mostly informed<br>1 Very informed
	15	elim_efforts_hcv_com	Additional comments:	notes
	16	elim_efforts_hbv	Do you consider the status of current elimination efforts for hepatitis B in your country to be:<br><br>(select more than one if relevant)	checkbox, Required<br>1 elim_efforts_hbv___1 On track to reach the WHO 2020 target of a hepatitis B antibody positive prevalence less than or equal to 1.0% among 5-year olds<br>2 elim_efforts_hbv___2 On track to reach the WHO 2030 target of a hepatitis B antibody positive prevalence less than or equal to 0.1% among 5-year olds<br>0 elim_efforts_hbv___0 WHO elimination targets in above options are unachievable given present policy

	17	elim_efforts_hbv_ci_mx<br><br>Show the field ONLY if: [elim_efforts_hbv(1)] = '1' or [elim_efforts_hbv(2)] = '1' or [elim_efforts_hbv(0)] = '1'	How informed do you consider yourself to be on the subject of this question?	radio (Matrix), Required<br>0 Not informed<br>3 Slightly informed<br>2 Mostly informed<br>1 Very informed
	18	elim_efforts_hbv_com	Additional comments:	notes<br>Custom alignment: RH
	19	policy_barriers_equity	Section Header: *Section 2. Enabling Environment and the WHO Global Health Sector Strategy*<br><br>The WHO Global Health Sector Strategy on Viral Hepatitis, 2016-20211 recommends removing "legal, regulatory, and policy barriers that hinder equitable access to hepatitis services" such as high-threshold harm reduction services, fibrosis level restrictions and drug abstinence policies for hepatitis C treatment.	descriptive
	20	policy_barriers_equity_mx_q	On a scale of 1-5 please indicate how well you think this recommendation is being implemented today in your country.	radio (Matrix), Required<br>0 Not implemented at all (1)<br>1 Somewhat implemented (2)<br>2 Moderately implemented (3)<br>3 Mostly implemented (4)<br>4 Fully implemented (5)
	21	policy_barriers_equity_mx_ci<br><br>Show the field ONLY if: [policy_barriers_equity_mx_q] = '0' or [policy_barriers_equity_mx_q] = '1' or [policy_barriers_equity_mx_q] = '2' or [policy_barriers_equity_mx_q] = '3' or [policy_barriers_equity_mx_q] = '4'	How informed do you consider yourself to be on the subject of this question?	radio (Matrix), Required<br>0 Not informed<br>3 Slightly informed<br>2 Mostly informed<br>1 Very informed
	22	policy_barriers_equity_com	Additional comments:	notes

	23	policy_stigma_discrim	The WHO Global Health Sector Strategy, 2016-20211 recommends to, "end policies and practices that condone or encourage stigma and discrimination against people at risk for hepatitis or living with hepatitis" such as employment discrimination based on disease status.	descriptive
	24	policy_stigma_discrim_mx_q	On a scale of 1-5, please indicate how well you think this recommendation is being implemented today in your country.	radio (Matrix), Required<br>0 Not implemented at all (1)<br>1 Somewhat implemented (2)<br>2 Moderately implemented (3)<br>3 Mostly implemented (4)<br>4 Fully implemented (5)
	25	policy_stig_discrim_ci_mx<br>Show the field ONLY if: [policy_stigma_discrim_mx_q] = '0' or [policy_stigma_discrim_mx_q] = '1' or [policy_stigma_discrim_mx_q] = '2' or [policy_stigma_discrim_mx_q] = '3' or [policy_stigma_discrim_mx_q] = '4'	How informed do you consider yourself to be on the subject of this question?	radio (Matrix), Required<br>0 Not informed<br>3 Slightly informed<br>2 Mostly informed<br>1 Very informed
	26	policy_stigma_discrim_com	Additional comments:	notes
	27	policy_hbv_hcvacc	The WHO Global Health Sector Strategy on Viral Hepatitis, 2016-20211 recommends that health workers be provided with free immunization against hepatitis B virus, where appropriate, as well as hepatitis B post-exposure prophylaxis as necessary.	descriptive
	28	policy_hbv_hcvacc_mx_q	On a scale of 1-5, please indicate how well you think this recommendation is being implemented today in your country.	radio (Matrix), Required<br>0 Not implemented at all (1)<br>1 Somewhat implemented (2)<br>2 Moderately implemented (3)<br>3 Mostly implemented (4)<br>4 Fully implemented (5)

	29	policy_hbv_hcvacc_ci_mx<br><br>Show the field ONLY if: [policy_hbv_hcvacc_mx_q] = '0' or [policy_hbv_hcvacc_mx_q] = '1' or [policy_hbv_hcvacc_mx_q] = '2' or [policy_hbv_hcvacc_mx_q] = '3' or [policy_hbv_hcvacc_mx_q] = '4'	How informed do you consider yourself to be on the subject of this question?	radio (Matrix), Required<br>0 Not informed<br>3 Slightly informed<br>2 Mostly informed<br>1 Very informed
	30	policy_hbv_hcvacc_com	Additional comments:	notes
	31	policy_hbv_strat	Section Header: *Section 3. National Strategy and/or Action Plan*<br><br>Does your country have a written national hepatitis B virus (HBV) strategy and/or action plan (or one that is part of a broader viral hepatitis strategy and/or action plan) that has been approved or ratified by the government?	radio, Required<br>1 Yes<br>0 No<br>99 Do not know
	32	policy_hbv_strat_comp<br><br>Show the field ONLY if: [policy_hbv_strat] = '1'	Below is a figure which illustrates the overall aspects a comprehensive viral hepatitis management framework should have, as set out in the WHO Global Hepatitis Report, 20172.	descriptive
	33	policy_hbv_strat_comp_mx_q<br><br>Show the field ONLY if: [policy_hbv_strat] = '1'	On a scale of 1-5, please indicate how comprehensive you believe your country's national strategy/plan for HBV is.	radio (Matrix), Required<br>0 Not comprehensive at all (1)<br>1 Somewhat comprehensive (2)<br>2 Moderately comprehensive (3)<br>3 Mostly comprehensive (4)<br>4 Completely comprehensive (5)
	34	policy_hbv_strat_improve<br><br>Show the field ONLY if: [policy_hbv_strat] = '1'	How well are the following areas addressed in the strategy/plan?	descriptive

	35	policy_hbv_strat_improve_mx_prev<br><br>Show the field ONLY if:<br>[policy_hbv_strat] = '1'	Prevention [1-5]	radio (Matrix), Required<br>0 Not addressed at all (1)<br>1 Somewhat addressed (2)<br>2 Moderately addressed (3)<br>3 Mostly addressed (4)<br>4 Completely addressed (5)
	36	policy_hbv_strat_improve_mx_test<br><br>Show the field ONLY if:<br>[policy_hbv_strat] = '1'	Testing [1-5]	radio (Matrix), Required<br>0 Not addressed at all (1)<br>1 Somewhat addressed (2)<br>2 Moderately addressed (3)<br>3 Mostly addressed (4)<br>4 Completely addressed (5)
	37	policy_hbv_strat_improve_mx_link<br><br>Show the field ONLY if:<br>[policy_hbv_strat] = '1'	Linkage to care [1-5]	radio (Matrix), Required<br>0 Not addressed at all (1)<br>1 Somewhat addressed (2)<br>2 Moderately addressed (3)<br>3 Mostly addressed (4)<br>4 Completely addressed (5)
	38	policy_hbv_strat_improve_mx_tx<br><br>Show the field ONLY if:<br>[policy_hbv_strat] = '1'	Treatment [1-5]	radio (Matrix), Required<br>0 Not addressed at all (1)<br>1 Somewhat addressed (2)<br>2 Moderately addressed (3)<br>3 Mostly addressed (4)<br>4 Completely addressed (5)

	39	policy_hbv_strat_improve_mx_care<br><br>Show the field ONLY if: [policy_hbv_strat] = '1'	Chronic care [1-5]	radio (Matrix), Required<br>0 Not addressed at all (1)<br>1 Somewhat addressed (2)<br>2 Moderately addressed (3)<br>3 Mostly addressed (4)<br>4 Completely addressed (5)
	40	policy_hbv_strat_ci_mx<br><br>Show the field ONLY if: [policy_hbv_strat] = '1' or [policy_hbv_strat] = '99' or [policy_hbv_strat] = '0'	How informed do you consider yourself to be on the subject of this question?	radio (Matrix), Required<br>0 Not informed<br>3 Slightly informed<br>2 Mostly informed<br>1 Very informed
	41	policy_hbv_strat_com	Additional comments:	notes
	42	policy_hcv_strat_1<br><br>Show the field ONLY if: [contact_info_country] = '2' or [contact_info_country] = '10' or [contact_info_country] = '11' or [contact_info_country] = '12' or [contact_info_country] = '14' or [contact_info_country] = '17' or [contact_info_country] = '20' or [contact_info_country] = '24' or [contact_info_country] = '25' or [contact_info_country] = '28' or [contact_info_country] = '29' or [contact_info_country] = '30'	In Summer 2018, your patient group reported that your country has a written national hepatitis C virus (HCV) strategy and/or action plan (or one that is part of a broader viral hepatitis strategy and/or action plan) that has been approved or ratified by the government. Is this correct?	radio, Required<br>1 My country has a written HCV strategy and/or action plan<br>0 My country does not have a written HCV strategy and/or action plan<br>99 Do not know

	43	policy_hcv_strat_0<br><br>Show the field ONLY if:<br>[contact_info_country] = '1' or [contact_info_country] = '3' or [contact_info_country] = '4' or [contact_info_country] = '5' or [contact_info_country] = '7' or [contact_info_country] = '16' or [contact_info_country] = '18' or [contact_info_country] = '19' or [contact_info_country] = '21' or [contact_info_country] = '22' or [contact_info_country] = '26'	In Summer 2018, your patient group reported that your country does not have a written national hepatitis C virus (HCV) strategy and/or action plan (or one that is part of a broader viral hepatitis strategy and/or action plan) that has been approved or ratified by the government. Is this correct?	radio, Required<br>1 My country has a written HCV strategy and/or action plan<br>0 My country does not have a written HCV strategy and/or action plan<br>99 Do not know
	44	policy_hcv_strat_99<br><br>Show the field ONLY if:<br>[contact_info_country] = '6' or [contact_info_country] = '9' or [contact_info_country] = '13' or [contact_info_country] = '15' or [contact_info_country] = '23'	In Summer 2018, your patient group did not report whether or not your country has a written national hepatitis C virus (HCV) strategy and/or action plan (or one that is part of a broader viral hepatitis strategy and/or action plan) that has been approved or ratified by the government.	radio, Required<br>1 My country has a written HCV strategy and/or action plan<br>0 My country does not have a written HCV strategy and/or action plan<br>99 Do not know
	45	policy_hcv_strat_comp<br><br>Show the field ONLY if:<br>[policy_hcv_strat_1] = '1' or [policy_hcv_strat_0] = '1' or [policy_hcv_strat_99] = '1'	Below is a figure which illustrates the overall aspects a comprehensive viral hepatitis management framework should have, as set out in the WHO Global Hepatitis Report, 20172.	descriptive
	46	policy_hcv_strat_comp_mx_q<br><br>Show the field ONLY if:<br>[policy_hcv_strat_1] = '1' or [policy_hcv_strat_0] = '1' or [policy_hcv_strat_99] = '1'	On a scale of 1-5, please indicate how comprehensive you believe your country's national strategy/plan for HCV is.	radio (Matrix), Required<br>0 Not comprehensive at all (1)<br>1 Somewhat comprehensive (2)<br>2 Moderately comprehensive (3)<br>3 Mostly comprehensive (4)<br>4 Completely comprehensive (5)

	47	policy_hcv_strat_improve<br><br>Show the field ONLY if:<br>[policy_hcv_strat_1] = '1' or [policy_hcv_strat_0] = '1' or [policy_hcv_strat_99] = '1'	Which areas do you think need to be better addressed in the strategy/plan:	descriptive
	48	policy_hcv_strat_improve_q1<br><br>Show the field ONLY if:<br>[policy_hcv_strat_1] = '1' or [policy_hcv_strat_0] = '1' or [policy_hcv_strat_99] = '1'	Prevention [1-5]	radio (Matrix), Required<br>0 Not addressed at all (1)<br>1 Somewhat addressed (2)<br>2 Moderately addressed (3)<br>3 Mostly addressed (4)<br>4 Completely addressed (5)
	49	policy_hcv_strat_improve_q2<br><br>Show the field ONLY if:<br>[policy_hcv_strat_1] = '1' or [policy_hcv_strat_0] = '1' or [policy_hcv_strat_99] = '1'	Testing [1-5]	radio (Matrix), Required<br>0 Not addressed at all (1)<br>1 Somewhat addressed (2)<br>2 Moderately addressed (3)<br>3 Mostly addressed (4)<br>4 Completely addressed (5)
	50	policy_hcv_strat_improve_q3<br><br>Show the field ONLY if:<br>[policy_hcv_strat_1] = '1' or [policy_hcv_strat_0] = '1' or [policy_hcv_strat_99] = '1'	Linkage to care [1-5]	radio (Matrix), Required<br>0 Not addressed at all (1)<br>1 Somewhat addressed (2)<br>2 Moderately addressed (3)<br>3 Mostly addressed (4)<br>4 Completely addressed (5)

	51	policy_hcv_strat_improve_q4<br><br>Show the field ONLY if:<br>[policy_hcv_strat_1] = '1' or [policy_hcv_strat_0] = '1' or [policy_hcv_strat_99] = '1'	Treatment [1-5]	radio (Matrix), Required<br>0 Not addressed at all (1)<br>1 Somewhat addressed (2)<br>2 Moderately addressed (3)<br>3 Mostly addressed (4)<br>4 Completely addressed (5)
	52	policy_hcv_strat_improve_q5<br><br>Show the field ONLY if:<br>[policy_hcv_strat_1] = '1' or [policy_hcv_strat_0] = '1' or [policy_hcv_strat_99] = '1'	Chronic care [1-5]	radio (Matrix), Required<br>0 Not addressed at all (1)<br>1 Somewhat addressed (2)<br>2 Moderately addressed (3)<br>3 Mostly addressed (4)<br>4 Completely addressed (5)
	53	policy_hcv_strat_improve_q6<br><br>Show the field ONLY if:<br>[policy_hcv_strat_1] = '1' or [policy_hcv_strat_0] = '1' or [policy_hcv_strat_99] = '1'	Re-treatment for those who do not achieve sustained virologic response (SVR) for HCV [1-5]	radio (Matrix), Required<br>0 Not addressed at all (1)<br>1 Somewhat addressed (2)<br>2 Moderately addressed (3)<br>3 Mostly addressed (4)<br>4 Completely addressed (5)
	54	policy_hcv_strat_ci_mx<br><br>Show the field ONLY if:<br>[policy_hcv_strat_1] = '1' or [policy_hcv_strat_1] = '0' or [policy_hcv_strat_1] = '99' or [policy_hcv_strat_0] = '1' or [policy_hcv_strat_0] = '0' or [policy_hcv_strat_0] = '99' or [policy_hcv_strat_99] = '1' or [policy_hcv_strat_99] = '0' or [policy_hcv_strat_99] = '99'	How informed do you consider yourself to be on the subject of this question?	radio (Matrix), Required<br>0 Not informed<br>3 Slightly informed<br>2 Mostly informed<br>1 Very informed
	55	policy_hcv_strat_com	Additional comments:	notes

	56	non_hospital_testing<br><br>Show the field ONLY if:<br>[contact_info_country] = '4' or [contact_info_country] = '7' or [contact_info_country] = '9' or [contact_info_country] = '10' or [contact_info_country] = '11' or [contact_info_country] = '14' or [contact_info_country] = '19' or [contact_info_country] = '20' or [contact_info_country] = '22' or [contact_info_country] = '24' or [contact_info_country] = '25' or [contact_info_country] = '27' or [contact_info_country] = '28' or [contact_info_country] = '30'	Section Header:<br>Your country allows for hepatitis C testing in non-hospital settings2. How well do you believe this is functioning in practice?<br><br>Please consider factors such as geographic reach (e.g. rural vs. urban) and diversity of testing location (e.g. migrant reception centres, prisons, harm reduction centres, non-governmental organizations (NGOs) etc.).	descriptive
	57	non_hospital_testing_mx_q<br><br>Show the field ONLY if:<br>[contact_info_country] = '4' or [contact_info_country] = '7' or [contact_info_country] = '9' or [contact_info_country] = '10' or [contact_info_country] = '11' or [contact_info_country] = '14' or [contact_info_country] = '19' or [contact_info_country] = '20' or [contact_info_country] = '22' or [contact_info_country] = '24' or [contact_info_country] = '25' or [contact_info_country] = '27' or [contact_info_country] = '28' or [contact_info_country] = '30'	On a scale of 1-5 how well do you believe this is functioning in practice?	radio (Matrix), Required<br>0 Not functioning at all in practice (1)<br>1 Slightly functioning in practice (2)<br>2 Moderately functioning in practice (3)<br>3 Mostly functioning in practice (4)<br>4 Fully functioning (5)

	58	non_hospital_testing_ci_mx<br><br>Show the field ONLY if:<br>[non_hospital_testing_mx_q] = '1' or [non_hospital_testing_mx_q] = '0' or [non_hospital_testing_mx_q] = '2' or [non_hospital_testing_mx_q] = '2' or [non_hospital_testing_mx_q] = '3' or [non_hospital_testing_mx_q] = '4'	How informed do you consider yourself to be on the subject of this question?	radio (Matrix), Required<br>0 Not informed<br>3 Slightly informed<br>2 Mostly informed<br>1 Very informed
	59	non_hospital_testing_com<br><br>Show the field ONLY if:<br>[contact_info_country] = '4' or [contact_info_country] = '7' or [contact_info_country] = '9' or [contact_info_country] = '10' or [contact_info_country] = '11' or [contact_info_country] = '14' or [contact_info_country] = '19' or [contact_info_country] = '20' or [contact_info_country] = '22' or [contact_info_country] = '24' or [contact_info_country] = '25' or [contact_info_country] = '27' or [contact_info_country] = '28' or [contact_info_country] = '30'	Additional comments:	notes
	60	non_hospital_treat<br><br>Show the field ONLY if:<br>[contact_info_country] = '8' or [contact_info_country] = '10' or [contact_info_country] = '11' or [contact_info_country] = '14' or [contact_info_country] = '21' or [contact_info_country] = '27' or [contact_info_country] = '30'	In 2017, your patient group reported that your country allows for hepatitis C treatment in non-hospital settings3. How well do you believe this is functioning in practice?<br><br>Please consider factors such as geographic reach (e.g. rural vs. urban) and diversity of testing location (e.g. migrant reception centres, prisons, harm reduction centres, NGO locations).	descriptive

	61	non_hospital_treat_mx_q<br><br>Show the field ONLY if:<br>[contact_info_country] = '8' or [contact_info_country] = '10' or [contact_info_country] = '11' or [contact_info_country] = '14' or [contact_info_country] = '21' or [contact_info_country] = '27' or [contact_info_country] = '30'	On a scale of 1-5, how well do you believe this is functioning in practice?	radio (Matrix), Required<br>0 Not functioning at all in practice (1)<br>1 Slightly functioning in practice (2)<br>2 Moderately functioning in practice (3)<br>3 Mostly functioning in practice (4)<br>4 Fully functioning (5)
	62	non_hospital_treat_ci_mx<br><br>Show the field ONLY if:<br>[non_hospital_treat_mx_q] = '0' or [non_hospital_treat_mx_q] = '1' or [non_hospital_treat_mx_q] = '2' or [non_hospital_treat_mx_q] = '3' or [non_hospital_treat_mx_q] = '4'	How informed do you consider yourself to be on the subject of this question?	radio (Matrix), Required<br>0 Not informed<br>3 Slightly informed<br>2 Mostly informed<br>1 Very informed
	63	non_hospital_treat_com<br><br>Show the field ONLY if:<br>[contact_info_country] = '8' or [contact_info_country] = '10' or [contact_info_country] = '11' or [contact_info_country] = '14' or [contact_info_country] = '21' or [contact_info_country] = '27' or [contact_info_country] = '30'	Additional comments:	notes
	64	non_special_treat<br><br>Show the field ONLY if:<br>[contact_info_country] = '11' or [contact_info_country] = '30'	Your country allows for hepatitis C treatment by non-specialists5. How well do you believe this is functioning in practice?<br><br>Please consider factors such as geographic reach (e.g. rural vs. urban) and diversity of testing location (e.g. migrant reception centres, prisons, harm reduction centres, NGO locations).	descriptive

	65	non_special_treat_mx_q<br><br>Show the field ONLY if: [contact_info_country] = '11' or [contact_info_country] = '30'	On a scale of 1-5, how well do you believe this is functioning in practice?	radio (Matrix), Required<br>0 Not functioning at all in practice (1)<br>1 Slightly functioning in practice (2)<br>2 Moderately functioning in practice (3)<br>3 Mostly functioning in practice (4)<br>4 Fully functioning (5)
	66	non_special_treat_ci_mx<br><br>Show the field ONLY if: [non_special_treat_mx_q] = '0' or [non_special_treat_mx_q] = '1' or [non_special_treat_mx_q] = '2' or [non_special_treat_mx_q] = '3' or [non_special_treat_mx_q] = '4'	How informed do you consider yourself to be on the subject of this question?	radio (Matrix), Required<br>0 Not informed<br>3 Slightly informed<br>2 Mostly informed<br>1 Very informed
	67	non_special_treat_com<br><br>Show the field ONLY if: [contact_info_country] = '11' or [contact_info_country] = '30'	Additional comments:	notes
	68	ghss_monitoring	Section Header: *Section 4. Priority groups for micro-elimination*<br><br>The WHO Global Health Sector Strategy on Viral Hepatitis, 2016-20211, recommends that national context (including risk factors, high prevalence populations, country settings, and sub-national distribution of viral hepatitis incidence) be used to guide the adaptation and implementation of hepatitis care.	descriptive
	69	ghss_monitoring_mx_q	On a scale of 1-5 please indicate how well you think this recommendation is being implemented today in your country.	radio (Matrix), Required<br>0 Not implemented at all (1)<br>1 Somewhat implemented (2)<br>2 Moderately implemented (3)<br>3 Mostly implemented (4)<br>4 Fully implemented (5)

	70	ghss_monitoring_ci_mx<br><br>Show the field ONLY if: [ghss_monitoring_mx_q] = '4' or [ghss_monitoring_mx_q] = '3' or [ghss_monitoring_mx_q] = '2' or [ghss_monitoring_mx_q] = '1' or [ghss_monitoring_mx_q] = '0'	How informed do you consider yourself to be on this subject?	radio (Matrix), Required<br>0 Not informed<br>3 Slightly informed<br>2 Mostly informed<br>1 Very informed
	71	ghss_monitoring_com	Additional comments:	notes
	72	ghss_hcv_integrate	Section Header: *Section 5. Multi-stakeholder/clinical collaboration and government engagement:*<br><br>The WHO Global Health Sector Strategy on Viral Hepatitis, 2016-20211 recommends that countries adapt hepatitis C services within their national setting to ensure that people who are already engaged in related services, such as harm reduction or HIV treatment, get tested and treated for hepatitis C. This recommendation is also intended to reinforce links between health services.<br><br>On a scale of 1-5, how well do you feel your country is integrating HCV care in the following services:	descriptive
	73	ghss_hcv_integrate_mx_q1	Alcohol use services	radio (Matrix), Required<br>0 Not at all integrated (1)<br>1 Slightly integrated (2)<br>2 Moderately integrated (3)<br>3 Mostly integrated (4)<br>4 Fully integrated (5)
	74	ghss_hcv_integrate_mx_q2	Blood safety	radio (Matrix), Required<br>0 Not at all integrated (1)<br>1 Slightly integrated (2)<br>2 Moderately integrated (3)<br>3 Mostly integrated (4)<br>4 Fully integrated (5)

	75	ghss_hcv_integrate_mx_q3	Cancer prevention and management	radio (Matrix), Required<br>0 Not at all integrated (1)<br>1 Slightly integrated (2)<br>2 Moderately integrated (3)<br>3 Mostly integrated (4)<br>4 Fully integrated (5)
	76	ghss_hcv_integrate_mx_q4	Haemodialysis centres	radio (Matrix), Required<br>0 Not at all integrated (1)<br>1 Slightly integrated (2)<br>2 Moderately integrated (3)<br>3 Mostly integrated (4)<br>4 Fully integrated (5)
	77	ghss_hcv_integrate_mx_q5	Harm reduction services	radio (Matrix), Required<br>0 Not at all integrated (1)<br>1 Slightly integrated (2)<br>2 Moderately integrated (3)<br>3 Mostly integrated (4)<br>4 Fully integrated (5)
	78	ghss_hcv_integrate_mx_q6	HIV treatment clinics	radio (Matrix), Required<br>0 Not at all integrated (1)<br>1 Slightly integrated (2)<br>2 Moderately integrated (3)<br>3 Mostly integrated (4)<br>4 Fully integrated (5)

	79	ghss_hcv_integrate_mx_q7	Migrant health services	radio (Matrix), Required<br>0 Not at all integrated (1)<br>1 Slightly integrated (2)<br>2 Moderately integrated (3)<br>3 Mostly integrated (4)<br>4 Fully integrated (5)
	80	ghss_hcv_integrate_mx_q8	Non-communicable disease prevention and management	radio (Matrix), Required<br>0 Not at all integrated (1)<br>1 Slightly integrated (2)<br>2 Moderately integrated (3)<br>3 Mostly integrated (4)<br>4 Fully integrated (5)
	81	ghss_hcv_integrate_mx_q9	Sexual and reproductive health (including STI clinics)	radio (Matrix), Required<br>0 Not at all integrated (1)<br>1 Slightly integrated (2)<br>2 Moderately integrated (3)<br>3 Mostly integrated (4)<br>4 Fully integrated (5)
	82	ghss_hcv_integrate_mx_q98	Other (please specify below)	radio (Matrix)<br>0 Not at all integrated (1)<br>1 Slightly integrated (2)<br>2 Moderately integrated (3)<br>3 Mostly integrated (4)<br>4 Fully integrated (5)

	83	ghss_hcv_integrate_98<br><br>Show the field ONLY if: [ghss_hcv_integrate_mx_q98] = '4' or [ghss_hcv_integrate_mx_q98] = '3' or [ghss_hcv_integrate_mx_q98] = '2' or [ghss_hcv_integrate_mx_q98] = '1' or [ghss_hcv_integrate_mx_q98] = '0'	Which other service(s):	text, Required
	84	ghss_hcv_integrate_ci_mx	How informed do you consider yourself to be on this subject?	radio (Matrix), Required<br>0 Not informed<br>3 Slightly informed<br>2 Mostly informed<br>1 Very informed
	85	ghss_hcv_integrate_com	Additional comments:	notes
	86	ghss_groups_melim	The WHO Global Health Sector Strategy on Viral Hepatitis, 2016-20211 recommends that countries adapt viral hepatitis services to ensure that they cater to different populations and settings within the national context.<br><br>On a scale of 1-5 how well do you feel your country is targeting the following groups:	descriptive
	87	ghss_groups_melim_mx_q1	Generational cohorts	radio (Matrix), Required<br>0 Not targeted at all (1)<br>1 Slightly targeted (2)<br>2 Moderately targeted (3)<br>3 Mostly targeted (4)<br>4 Well targeted (5)

	88	ghss_groups_melim_mx_q2	Haemodialysis patients	radio (Matrix), Required<br>0 Not targeted at all (1)<br>1 Slightly targeted (2)<br>2 Moderately targeted (3)<br>3 Mostly targeted (4)<br>4 Well targeted (5)
	89	ghss_groups_melim_mx_q3	Haemophilia patients	radio (Matrix), Required<br>0 Not targeted at all (1)<br>1 Slightly targeted (2)<br>2 Moderately targeted (3)<br>3 Mostly targeted (4)<br>4 Well targeted (5)
	90	ghss_groups_melim_mx_q4	Men who have sex with men	radio (Matrix), Required<br>0 Not targeted at all (1)<br>1 Slightly targeted (2)<br>2 Moderately targeted (3)<br>3 Mostly targeted (4)<br>4 Well targeted (5)
	91	ghss_groups_melim_mx_q5	Migrants from countries with a high-prevalence of viral hepatitis	radio (Matrix), Required<br>0 Not targeted at all (1)<br>1 Slightly targeted (2)<br>2 Moderately targeted (3)<br>3 Mostly targeted (4)<br>4 Well targeted (5)

	92	ghss_groups_melim_mx_q6	Patients with advanced liver disease	radio (Matrix), Required<br>0 Not targeted at all (1)<br>1 Slightly targeted (2)<br>2 Moderately targeted (3)<br>3 Mostly targeted (4)<br>4 Well targeted (5)
	93	ghss_groups_melim_mx_q7	People living with HIV	radio (Matrix), Required<br>0 Not targeted at all (1)<br>1 Slightly targeted (2)<br>2 Moderately targeted (3)<br>3 Mostly targeted (4)<br>4 Well targeted (5)
	94	ghss_groups_melim_mx_q8	People who inject drugs	radio (Matrix), Required<br>0 Not targeted at all (1)<br>1 Slightly targeted (2)<br>2 Moderately targeted (3)<br>3 Mostly targeted (4)<br>4 Well targeted (5)
	95	ghss_groups_melim_mx_q9	Prisoners	radio (Matrix), Required<br>0 Not targeted at all (1)<br>1 Slightly targeted (2)<br>2 Moderately targeted (3)<br>3 Mostly targeted (4)<br>4 Well targeted (5)

	96	ghss_groups_melim_mx_q10	Sex workers	radio (Matrix), Required<br>0 Not targeted at all (1)<br>1 Slightly targeted (2)<br>2 Moderately targeted (3)<br>3 Mostly targeted (4)<br>4 Well targeted (5)
	97	ghss_groups_melim_mx_q11	Thalassemia patients	radio (Matrix), Required<br>0 Not targeted at all (1)<br>1 Slightly targeted (2)<br>2 Moderately targeted (3)<br>3 Mostly targeted (4)<br>4 Well targeted (5)
	98	ghss_groups_melim_mx_q12	Transgender people	radio (Matrix), Required<br>0 Not targeted at all (1)<br>1 Slightly targeted (2)<br>2 Moderately targeted (3)<br>3 Mostly targeted (4)<br>4 Well targeted (5)
	99	ghss_groups_melim_mx_q13	Transplant patients	radio (Matrix), Required<br>0 Not targeted at all (1)<br>1 Slightly targeted (2)<br>2 Moderately targeted (3)<br>3 Mostly targeted (4)<br>4 Well targeted (5)

	100	ghss_groups_melim_mx_q14	Veteran/Military personnel	radio (Matrix), Required<br>0 Not targeted at all (1)<br>1 Slightly targeted (2)<br>2 Moderately targeted (3)<br>3 Mostly targeted (4)<br>4 Well targeted (5)
	101	ghss_groups_melim_mx_q98	Other (please specify below)	radio (Matrix)<br>0 Not targeted at all (1)<br>1 Slightly targeted (2)<br>2 Moderately targeted (3)<br>3 Mostly targeted (4)<br>4 Well targeted (5)
	102	ghss_groups_melim_98<br><br>Show the field ONLY if: [ghss_groups_melim_mx_q98] = '0' or [ghss_groups_melim_mx_q98] = '1' or [ghss_groups_melim_mx_q98] = '2' or [ghss_groups_melim_mx_q98] = '3' or [ghss_groups_melim_mx_q98] = '4'	Which other risk group(s):	text, Required
	103	ghss_groups_melim_ci_mx	How informed do you consider yourself to be on this subject?	radio (Matrix), Required<br>0 Not informed<br>3 Slightly informed<br>2 Mostly informed<br>1 Very informed
	104	ghss_groups_melim_com	Additional comments:	notes

	105	treat_restrict_fib<br><br>Show the field ONLY if:<br>[contact_info_country] = '1' or [contact_info_country] = '4' or [contact_info_country] = '7' or [contact_info_country] = '9' or [contact_info_country] = '10' or [contact_info_country] = '11' or [contact_info_country] = '15' or [contact_info_country] = '17' or [contact_info_country] = '18' or [contact_info_country] = '19' or [contact_info_country] = '20' or [contact_info_country] = '25' or [contact_info_country] = '26' or [contact_info_country] = '27' or [contact_info_country] = '30'	Section Header: *Section 6. Treatment Restrictions*<br><br>A recent review6 of the direct-acting antiviral (DAA) access policies across Europe revealed that your country does not restrict access to DAA therapy for hepatitis C virus based on fibrosis stage.	descriptive
	106	treat_restrict_fib_mx_q<br><br>Show the field ONLY if:<br>[contact_info_country] = '1' or [contact_info_country] = '4' or [contact_info_country] = '7' or [contact_info_country] = '9' or [contact_info_country] = '10' or [contact_info_country] = '11' or [contact_info_country] = '15' or [contact_info_country] = '17' or [contact_info_country] = '18' or [contact_info_country] = '19' or [contact_info_country] = '20' or [contact_info_country] = '25' or [contact_info_country] = '26' or [contact_info_country] = '27' or [contact_info_country] = '30'	On a scale of 1-5 how well overall do you believe this is functioning in practice?	radio (Matrix), Required<br>0 Not functioning at all in practice (1)<br>1 Slightly functioning in practice (2)<br>2 Moderately functioning in practice (3)<br>3 Mostly functioning in practice (4)<br>4 Fully functioning (5)

	107	treat_restrict_fib_ci_mx<br><br>Show the field ONLY if: [treat_restrict_fib_mx_q] = '0' or [treat_restrict_fib_mx_q] = '1' or [treat_restrict_fib_mx_q] = '2' or [treat_restrict_fib_mx_q] = '3' or [treat_restrict_fib_mx_q] = '4'	How informed do you consider yourself to be on this subject?	radio (Matrix), Required<br>0 Not informed<br>3 Slightly informed<br>2 Mostly informed<br>1 Very informed
	108	treat_restrict_fib_com<br><br>Show the field ONLY if: [contact_info_country] = '1' or [contact_info_country] = '4' or [contact_info_country] = '7' or [contact_info_country] = '9' or [contact_info_country] = '10' or [contact_info_country] = '11' or [contact_info_country] = '15' or [contact_info_country] = '17' or [contact_info_country] = '18' or [contact_info_country] = '19' or [contact_info_country] = '20' or [contact_info_country] = '25' or [contact_info_country] = '26' or [contact_info_country] = '27' or [contact_info_country] = '30'	Additional comments:	notes

	109	drug_alc_restrict_0<br><br>Show the field ONLY if: [contact_info_country] = '1' or [contact_info_country] = '2' or [contact_info_country] = '7' or [contact_info_country] = '9' or [contact_info_country] = '10' or [contact_info_country] = '11' or [contact_info_country] = '12' or [contact_info_country] = '15' or [contact_info_country] = '17' or [contact_info_country] = '18' or [contact_info_country] = '19' or [contact_info_country] = '20' or [contact_info_country] = '24' or [contact_info_country] = '25' or [contact_info_country] = '26' or [contact_info_country] = '30'	A recent review5 of the direct-acting antiviral (DAA) access policies across Europe revealed that your country does not restrict DAA therapy for HCV treatment based on alcohol use and/or drug use.	descriptive<br>1 General population 2, High-risk population(s) (please specify below) 0, None of the above 99, Do not know
	110	drug_alc_restrict_0_mx_q<br><br>Show the field ONLY if: [contact_info_country] = '1' or [contact_info_country] = '2' or [contact_info_country] = '7' or [contact_info_country] = '9' or [contact_info_country] = '10' or [contact_info_country] = '11' or [contact_info_country] = '12' or [contact_info_country] = '15' or [contact_info_country] = '17' or [contact_info_country] = '18' or [contact_info_country] = '19' or [contact_info_country] = '20' or [contact_info_country] = '24' or [contact_info_country] = '25' or [contact_info_country] = '26' or [contact_info_country] = '30'	On a scale of 1-5 how well overall do you believe this is functioning in practice?	radio (Matrix), Required<br>0 Not functioning at all in practice (1)<br>1 Slightly functioning in practice (2)<br>2 Moderately functioning in practice (3)<br>3 Mostly functioning in practice (4)<br>4 Fully functioning (5)

	111	drug_alc_restrict_1<br><br>Show the field ONLY if:<br>[contact_info_country] = '4' or [contact_info_country] = '5' or [contact_info_country] = '6' or [contact_info_country] = '13' or [contact_info_country] = '2 3'	A recent review5 of the direct-acting antiviral (DAA) access policies across Europe revealed that your country has additional restrictions on DAA therapy for HCV treatment based on alcohol use and/or drug use.	descriptive<br>1 | General population 2, High-risk population(s) (please specify below) 0, None of the above 99, Do not know
	112	drug_alc_restrict_1_mx_q<br><br>Show the field ONLY if:<br>[contact_info_country] = '4' or [contact_info_country] = '5' or [contact_info_country] = '6' or [contact_info_country] = '13' or [contact_info_country] = '2 3'	On a scale of 1-5 how well overall do you believe this is functioning in practice?	radio (Matrix), Required<br>0 | Not functioning at all in practice (1)<br>1 | Slightly functioning in practice (2)<br>2 | Moderately functioning in practice (3)<br>3 | Mostly functioning in practice (4)<br>4 | Fully functioning (5)
	113	drug_alc_restrict_2<br><br>Show the field ONLY if:<br>[contact_info_country] = '27'	A recent review5 of the direct-acting antiviral (DAA) access policies across Europe revealed that your country prioritized DAA therapy for HCV treatment based on alcohol use and/or drug use.	descriptive<br>1 | General population 2, High-risk population(s) (please specify below) 0, None of the above 99, Do not know
	114	drug_alc_restrict_2_mx_q<br><br>Show the field ONLY if:<br>[contact_info_country] = '27'	On a scale of 1-5 how well overall do you believe this is functioning in practice?	radio (Matrix), Required<br>0 | Not functioning at all in practice (1)<br>1 | Slightly functioning in practice (2)<br>2 | Moderately functioning in practice (3)<br>3 | Mostly functioning in practice (4)<br>4 | Fully functioning (5)
	115	drug_alc_restrict_98<br><br>Show the field ONLY if:<br>[contact_info_country] = '21'	A recent review5 of the direct-acting antiviral (DAA) access policies across Europe revealed that your country requires patients to have a negative drug test in order to have access to DAA therapy for HCV treatment based on alcohol use and/or drug use.	descriptive<br>1 | General population 2, High-risk population(s) (please specify below) 0, None of the above 99, Do not know

	116	drug_alc_restrict_98_mx_q<br><br>Show the field ONLY if: [contact_info_country] = '21'	On a scale of 1-5 how well overall do you believe this is functioning in practice?	radio (Matrix), Required<br>0 Not functioning at all in practice (1)<br>1 Slightly functioning in practice (2)<br>2 Moderately functioning in practice (3)<br>3 Mostly functioning in practice (4)<br>4 Fully functioning (5)
	117	drug_alc_restrict_ci_mx<br><br>Show the field ONLY if: [contact_info_country] = '1' or [contact_info_country] = '2' or [contact_info_country] = '4' or [contact_info_country] = '5' or [contact_info_country] = '6' or [contact_info_country] = '7' or [contact_info_country] = '9' or [contact_info_country] = '10' or [contact_info_country] = '11' or [contact_info_country] = '12' or [contact_info_country] = '13' or [contact_info_country] = '15' or [contact_info_country] = '17' or [contact_info_country] = '18' or [contact_info_country] = '19' or [contact_info_country] = '20' or [contact_info_country] = '21' or [contact_info_country] = '23' or [contact_info_country] = '24' or [contact_info_country] = '25' or [contact_info_country] = '26' or [contact_info_country] = '27' or [contact_info_country] = '30'	How informed do you consider yourself to be on this subject?	radio (Matrix), Required<br>0 Not informed<br>3 Slightly informed<br>2 Mostly informed<br>1 Very informed

	118	drug_alc_restriction_com<br><br>Show the field ONLY if: [contact_info_country] = '1' or [contact_info_country] = '2' or [contact_info_country] = '4' or [contact_info_country] = '5' or [contact_info_country] = '6' or [contact_info_country] = '7' or [contact_info_country] = '9' or [contact_info_country] = '10' or [contact_info_country] = '11' or [contact_info_country] = '12' or [contact_info_country] = '13' or [contact_info_country] = '15' or [contact_info_country] = '17' or [contact_info_country] = '18' or [contact_info_country] = '19' or [contact_info_country] = '20' or [contact_info_country] = '21' or [contact_info_country] = '23' or [contact_info_country] = '24' or [contact_info_country] = '25' or [contact_info_country] = '26' or [contact_info_country] = '27' or [contact_info_country] = '30'	Additional comments:	notes

	119	nsp_genpop<br><br>Show the field ONLY if:<br>[contact_info_country] = '1' or [contact_info_country] = '2' or [contact_info_country] = '4' or [contact_info_country] = '5' or [contact_info_country] = '6' or [contact_info_country] = '7' or [contact_info_country] = '9' or [contact_info_country] = '10' or [contact_info_country] = '11' or [contact_info_country] = '12' or [contact_info_country] = '13' or [contact_info_country] = '15' or [contact_info_country] = '17' or [contact_info_country] = '18' or [contact_info_country] = '19' or [contact_info_country] = '20' or [contact_info_country] = '21' or [contact_info_country] = '23' or [contact_info_country] = '24' or [contact_info_country] = '25' or [contact_info_country] = '26' or [contact_info_country] = '29' or [contact_info_country] = '30' or [contact_info_country] = '22' or [contact_info_country] = '14' or [contact_info_country] = '3'	Section Header: *Section 7. Harm Reduction Services*<br>According to the 2017 EMCDDA Drug Report7, your country provides clean injecting equipment free of charge in the general population. Please indicate how well you believe this is functioning in practice in your country.<br><br>Please consider factors such as geographic reach (e.g. availability in non-urban areas) and opening hours.	descriptive

	120	nsp_genpop_mx_q<br><br>Show the field ONLY if: [contact_info_country] = '1' or [contact_info_country] = '2' or [contact_info_country] = '4' or [contact_info_country] = '5' or [contact_info_country] = '6' or [contact_info_country] = '7' or [contact_info_country] = '9' or [contact_info_country] = '10' or [contact_info_country] = '11' or [contact_info_country] = '12' or [contact_info_country] = '13' or [contact_info_country] = '15' or [contact_info_country] = '17' or [contact_info_country] = '18' or [contact_info_country] = '19' or [contact_info_country] = '20' or [contact_info_country] = '21' or [contact_info_country] = '23' or [contact_info_country] = '24' or [contact_info_country] = '25' or [contact_info_country] = '26' or [contact_info_country] = '29' or [contact_info_country] = '30' or [contact_info_country] = '22' or [contact_info_country] = '14' or [contact_info_country] = '3'	On a scale of 1-5, please indicate how well you believe this is functioning in practice in your country.	radio (Matrix)<br>0 Not functioning at all in practice (1)<br>1 Slightly functioning in practice (2)<br>2 Moderately functioning in practice (3)<br>3 Mostly functioning in practice (4)<br>4 Fully functioning (5)
	121	nsp_genpop_ci_mx<br><br>Show the field ONLY if: [nsp_genpop_mx_q] = '0' or [nsp_genpop_mx_q] = '1' or [nsp_genpop_mx_q] = '2' or [nsp_genpop_mx_q] = '3' or [nsp_genpop_mx_q] = '4'	How informed do you consider yourself to be on the subject of this question?	radio (Matrix), Required<br>0 Not informed<br>3 Slightly informed<br>2 Mostly informed<br>1 Very informed

	122	nsp_genpop_com<br><br>Show the field ONLY if:<br>[contact_info_country] = '1' or [contact_info_country] = '2' or [contact_info_country] = '4' or [contact_info_country] = '5' or [contact_info_country] = '6' or [contact_info_country] = '7' or [contact_info_country] = '9' or [contact_info_country] = '10' or [contact_info_country] = '11' or [contact_info_country] = '12' or [contact_info_country] = '13' or [contact_info_country] = '15' or [contact_info_country] = '17' or [contact_info_country] = '18' or [contact_info_country] = '19' or [contact_info_country] = '20' or [contact_info_country] = '21' or [contact_info_country] = '23' or [contact_info_country] = '24' or [contact_info_country] = '25' or [contact_info_country] = '26' or [contact_info_country] = '29' or [contact_info_country] = '30' or [contact_info_country] = '22' or [contact_info_country] = '14' or [contact_info_country] = '3'	Additional comments:	notes

	123	ost_genpop<br><br>Show the field ONLY if:<br>[contact_info_country] = '1' or [contact_info_country] = '2' or [contact_info_country] = '4' or [contact_info_country] = '5' or [contact_info_country] = '6' or [contact_info_country] = '7' or [contact_info_country] = '9' or [contact_info_country] = '10' or [contact_info_country] = '11' or [contact_info_country] = '12' or [contact_info_country] = '13' or [contact_info_country] = '15' or [contact_info_country] = '17' or [contact_info_country] = '18' or [contact_info_country] = '19' or [contact_info_country] = '20' or [contact_info_country] = '21' or [contact_info_country] = '23' or [contact_info_country] = '24' or [contact_info_country] = '25' or [contact_info_country] = '26' or [contact_info_country] = '29' or [contact_info_country] = '30' or [contact_info_country] = '22' or [contact_info_country] = '14' or [contact_info_country] = '3'	According to the 2017 EMCDDA Drug Report7 your country provides opioid substitution therapy (OST) in the general population. Please indicate how well you believe this is functioning in practice.<br><br>Please consider factors such as restrictions to OST based on other requirements such as abstinence from drug use.	descriptive

	124	ost_genpop_mx_q<br><br>Show the field ONLY if: [contact_info_country] = '1' or [contact_info_country] = '2' or [contact_info_country] = '4' or [contact_info_country] = '5' or [contact_info_country] = '6' or [contact_info_country] = '7' or [contact_info_country] = '9' or [contact_info_country] = '10' or [contact_info_country] = '11' or [contact_info_country] = '12' or [contact_info_country] = '13' or [contact_info_country] = '15' or [contact_info_country] = '17' or [contact_info_country] = '18' or [contact_info_country] = '19' or [contact_info_country] = '20' or [contact_info_country] = '21' or [contact_info_country] = '23' or [contact_info_country] = '24' or [contact_info_country] = '25' or [contact_info_country] = '26' or [contact_info_country] = '29' or [contact_info_country] = '30' or [contact_info_country] = '22' or [contact_info_country] = '14' or [contact_info_country] = '3'	On a scale of 1-5 please indicate how well you believe this is functioning in practice.	radio (Matrix), Required<br>0 Not functioning at all in practice (1)<br>1 Slightly functioning in practice (2)<br>2 Moderately functioning in practice (3)<br>3 Mostly functioning in practice (4)<br>4 Fully functioning (5)
	125	ost_genpop_ci_mx<br><br>Show the field ONLY if: [ost_genpop_mx_q] = '0' or [ost_genpop_mx_q] = '1' or [ost_genpop_mx_q] = '2' or [ost_genpop_mx_q] = '3' or [ost_genpop_mx_q] = '4'	How informed do you consider yourself to be on the subject of this question?	radio (Matrix), Required<br>0 Not informed<br>3 Slightly informed<br>2 Mostly informed<br>1 Very informed

	126	ost_genpop_com<br><br>Show the field ONLY if: [contact_info_country] = '1' or [contact_info_country] = '2' or [contact_info_country] = '4' or [contact_info_country] = '5' or [contact_info_country] = '6' or [contact_info_country] = '7' or [contact_info_country] = '9' or [contact_info_country] = '10' or [contact_info_country] = '11' or [contact_info_country] = '12' or [contact_info_country] = '13' or [contact_info_country] = '15' or [contact_info_country] = '17' or [contact_info_country] = '18' or [contact_info_country] = '19' or [contact_info_country] = '20' or [contact_info_country] = '21' or [contact_info_country] = '23' or [contact_info_country] = '24' or [contact_info_country] = '25' or [contact_info_country] = '26' or [contact_info_country] = '29' or [contact_info_country] = '30' or [contact_info_country] = '22' or [contact_info_country] = '14' or [contact_info_country] = '3'	Additional comments:	notes
	127	nsp_prison_1<br><br>Show the field ONLY if: [contact_info_country] = '25'	Section Header: *Section 8. Harm reduction, testing, and treatment in prison settings*<br><br>According to a recent review8 of the state of viral hepatitis care in European prisons, your country provides needle and syringe programmes (NSPs) in prisons.	descriptive

	128	nsp_prison_1_mx_q<br><br>Show the field ONLY if:<br>[contact_info_country] = '25'	On a scale of 1-5, please indicate how well you believe this is functioning in practice.	radio (Matrix), Required<br>0 Not functioning at all in practice (1)<br>1 Slightly functioning in practice (2)<br>2 Moderately functioning in practice (3)<br>3 Mostly functioning in practice (4)<br>4 Fully functioning (5)
	129	nsp_prison_2<br><br>Show the field ONLY if:<br>[contact_info_country] = '21'	According to a recent review8 of the state of viral hepatitis care in European prisons, your country provides needle and syringe programmes (NSPs) in prisons and needle exchange is technically available but is limited due to extra requirements.	descriptive
	130	nsp_prison_2_mx_q<br><br>Show the field ONLY if:<br>[contact_info_country] = '21'	On a scale of 1-5, please indicate how well you believe this is functioning in practice.	radio (Matrix), Required<br>0 Not functioning at all in practice (1)<br>1 Slightly functioning in practice (2)<br>2 Moderately functioning in practice (3)<br>3 Mostly functioning in practice (4)<br>4 Fully functioning (5)
	131	nsp_prison_3<br><br>Show the field ONLY if:<br>[contact_info_country] = '11' or [contact_info_country] = '1 6'	According to a recent review8 of the state of viral hepatitis care in European prisons, your country provides needle and syringe programmes (NSPs) in at least one prison.	descriptive
	132	nsp_prison_3_mx_q<br><br>Show the field ONLY if:<br>[contact_info_country] = '11' or [contact_info_country] = '1 6'	On a scale of 1-5, please indicate how well you believe this is functioning in practice.	radio (Matrix), Required<br>0 Not functioning at all in practice (1)<br>1 Slightly functioning in practice (2)<br>2 Moderately functioning in practice (3)<br>3 Mostly functioning in practice (4)<br>4 Fully functioning (5)

	133	nsp_prison_ci_mx<br><br>Show the field ONLY if: [contact_info_country] = '1' or [contact_info_country] = '2' or [contact_info_country] = '3' or [contact_info_country] = '4' or [contact_info_country] = '5' or [contact_info_country] = '7' or [contact_info_country] = '9' or [contact_info_country] = '10' or [contact_info_country] = '12' or [contact_info_country] = '13' or [contact_info_country] = '15' or [contact_info_country] = '17' or [contact_info_country] = '19' or [contact_info_country] = '20' or [contact_info_country] = '22' or [contact_info_country] = '23' or [contact_info_country] = '24' or [contact_info_country] = '26' or [contact_info_country] = '28' or [contact_info_country] = '29' or [contact_info_country] = '30' or [contact_info_country] = '11' or [contact_info_country] = '16'	How informed do you consider yourself to be on the subject of this question?	radio (Matrix), Required<br>0 Not informed<br>3 Slightly informed<br>2 Mostly informed<br>1 Very informed

	134	nsp_prison_com<br><br>Show the field ONLY if: [contact_info_country] = '1' or [contact_info_country] = '2' or [contact_info_country] = '3' or [contact_info_country] = '4' or [contact_info_country] = '5' or [contact_info_country] = '7' or [contact_info_country] = '9' or [contact_info_country] = '10' or [contact_info_country] = '12' or [contact_info_country] = '13' or [contact_info_country] = '15' or [contact_info_country] = '17' or [contact_info_country] = '19' or [contact_info_country] = '20' or [contact_info_country] = '22' or [contact_info_country] = '23' or [contact_info_country] = '24' or [contact_info_country] = '26' or [contact_info_country] = '28' or [contact_info_country] = '29' or [contact_info_country] = '30' or [contact_info_country] = '11' or [contact_info_country] = '16' or [contact_info_country] = '21' or [contact_info_country] = '25'	Additional comments:	notes

	135	ost_prison_1<br><br>Show the field ONLY if: [contact_info_country] = '1' or [contact_info_country] = '2' or [contact_info_country] = '5' or [contact_info_country] = '10' or [contact_info_country] = '15' or [contact_info_country] = '16' or [contact_info_country] = '20' or [contact_info_country] = '21' or [contact_info_country] = '24' or [contact_info_country] = '25' or [contact_info_country] = '30'	According to a recent review8 of the state of viral hepatitis care in European prisons your country provides opioid substitution therapy (OST) in prisons. Please indicate how well you believe this is functioning in practice in your country.<br><br>Consider factors such as restrictions to OST based on additional requirements such as abstinence from drug use, time of initiation, continuation of OST if previously prescribed in the community etc.	descriptive
	136	ost_prison_1_mx_q<br><br>Show the field ONLY if: [contact_info_country] = '1' or [contact_info_country] = '2' or [contact_info_country] = '5' or [contact_info_country] = '10' or [contact_info_country] = '15' or [contact_info_country] = '16' or [contact_info_country] = '20' or [contact_info_country] = '21' or [contact_info_country] = '24' or [contact_info_country] = '25' or [contact_info_country] = '30'	On a scale of 1-5 please indicate how well you believe this is functioning in practice in your country.	radio (Matrix), Required<br>0 Not functioning at all in practice (1)<br>1 Slightly functioning in practice (2)<br>2 Moderately functioning in practice (3)<br>3 Mostly functioning in practice (4)<br>4 Fully functioning (5)
	137	ost_prison_2<br><br>Show the field ONLY if: [contact_info_country] = '19'	According to a recent review8 of the state of viral hepatitis care in European prisons your country provides opioid substitution therapy (OST) in prisons, but extra requirements limit coverage. Please indicate how well you believe this is functioning in practice in your country.<br><br>Consider factors such as restrictions to OST based on additional requirements such as abstinence from drug use, time of initiation, continuation of OST if previously prescribed in the community etc.	descriptive

	138	ost_prison_2_mx_q<br><br>Show the field ONLY if: [contact_info_country] = '19'	On a scale of 1-5 please indicate how well you believe this is functioning in practice in your country.	radio (Matrix), Required<br>0 Not functioning at all in practice (1)<br>1 Slightly functioning in practice (2)<br>2 Moderately functioning in practice (3)<br>3 Mostly functioning in practice (4)<br>4 Fully functioning (5)
	139	ost_prison_3<br><br>Show the field ONLY if: [contact_info_country] = '7' or [contact_info_country] = '9' or [contact_info_country] = '17' or [contact_info_country] = '2 2'	According to a recent review8 of the state of viral hepatitis care in European prisons your country provides opioid substitution therapy (OST) in prisons only if it was started prior to incarceration. Please indicate how well you believe this is functioning in practice in your country.<br><br>Consider factors such as restrictions to OST based on additional requirements such as abstinence from drug use, time of initiation, continuation of OST if previously prescribed in the community etc.	descriptive
	140	ost_prison_3_mx_q<br><br>Show the field ONLY if: [contact_info_country] = '7' or [contact_info_country] = '9' or [contact_info_country] = '17' or [contact_info_country] = '2 2'	On a scale of 1-5 please indicate how well you believe this is functioning in practice in your country.	radio (Matrix), Required<br>0 Not functioning at all in practice (1)<br>1 Slightly functioning in practice (2)<br>2 Moderately functioning in practice (3)<br>3 Mostly functioning in practice (4)<br>4 Fully functioning (5)
	141	ost_prison_4<br><br>Show the field ONLY if: [contact_info_country] = '4' or [contact_info_country] = '11' or [contact_info_country] = '1 2' or [contact_info_country] = '13' or [contact_info_country] = '26'	According to a recent review8 of the state of viral hepatitis care in European prisons, opioid substitution therapy (OST) is available in some prisons in your country. Please indicate how well you believe this is functioning in practice in your country.<br><br>Consider factors such as restrictions to OST based on additional requirements such as abstinence from drug use, time of initiation, continuation of OST if previously prescribed in the community etc.	descriptive

	142	ost_prison_4_mx_q<br><br>Show the field ONLY if:<br>[contact_info_country] = '4' or [contact_info_country] = '11' or [contact_info_country] = '1 2' or [contact_info_country] = '13' or [contact_info_country] = '26'	On a scale of 1-5 please indicate how well you believe this is functioning in practice in your country.	radio (Matrix), Required<br>0 Not functioning at all in practice (1)<br>1 Slightly functioning in practice (2)<br>2 Moderately functioning in practice (3)<br>3 Mostly functioning in practice (4)<br>4 Fully functioning (5)
	143	ost_prison_ci_mx<br><br>Show the field ONLY if:<br>[ost_prison_1_mx_q] = '0' or [ost_prison_1_mx_q] = '1' or [ost_prison_1_mx_q] = '2' or [ost_prison_1_mx_q] = '4' or [ost_prison_2_mx_q] = '0' or [ost_prison_2_mx_q] = '1' or [ost_prison_2_mx_q] = '2' or [ost_prison_2_mx_q] = '3' or [ost_prison_2_mx_q] = '4' or [ost_prison_3_mx_q] = '0' or [ost_prison_3_mx_q] = '1' or [ost_prison_3_mx_q] = '2' or [ost_prison_3_mx_q] = '3' or [ost_prison_3_mx_q] = '4' or [ost_prison_4_mx_q] = '0' or [ost_prison_4_mx_q] = '1' or [ost_prison_4_mx_q] = '2' or [ost_prison_4_mx_q] = '3' or [ost_prison_4_mx_q] = '4'	How informed do you consider yourself to be on the subject of this question?	radio (Matrix), Required<br>0 Not informed<br>3 Slightly informed<br>2 Mostly informed<br>1 Very informed

	144	ost_prison_com<br><br>Show the field ONLY if:<br>[ost_prison_1_mx_q] = '0' or<br>[ost_prison_1_mx_q] = '1' or<br>[ost_prison_1_mx_q] = '2' or<br>[ost_prison_1_mx_q] = '4' or<br>[ost_prison_2_mx_q] = '0' or<br>[ost_prison_2_mx_q] = '1' or<br>[ost_prison_2_mx_q] = '2' or<br>[ost_prison_2_mx_q] = '3' or<br>[ost_prison_2_mx_q] = '4' or<br>[ost_prison_3_mx_q] = '0' or<br>[ost_prison_3_mx_q] = '1' or<br>[ost_prison_3_mx_q] = '2' or<br>[ost_prison_3_mx_q] = '3' or<br>[ost_prison_3_mx_q] = '4' or<br>[ost_prison_4_mx_q] = '0' or<br>[ost_prison_4_mx_q] = '1' or<br>[ost_prison_4_mx_q] = '2' or<br>[ost_prison_4_mx_q] = '3' or<br>[ost_prison_4_mx_q] = '4'	Additional comments:	notes
	145	test_screen_prison_1<br><br>Show the field ONLY if:<br>[contact_info_country] = '30'	A recent review8 of the state of viral hepatitis care in European prisons found that your country provides testing and/or universal screening for HCV in all prisons in the country.	descriptive, Required
	146	test_screen_prison_1_mx_q<br><br>Show the field ONLY if:<br>[contact_info_country] = '30'	On a scale of 1-5, please indicate how well you believe this is functioning in practice in prisons in your country.	radio (Matrix), Required<br>0 Not functioning at all in practice (1)<br>1 Slightly functioning in practice (2)<br>2 Moderately functioning in practice (3)<br>3 Mostly functioning in practice (4)<br>4 Fully functioning (5)

	147	test_screen_prison_2<br><br>Show the field ONLY if:<br>[contact_info_country] = '4' or [contact_info_country] = '5' or [contact_info_country] = '7' or [contact_info_country] = '10' or [contact_info_country] = '13' or [contact_info_country] = '17' or [contact_info_country] = '24' or [contact_info_country] = '25'	A recent review8 of the state of viral hepatitis care in European prisons found that your country provides testing and/or screening for HCV in prisons; however, the extent of coverage is unknown despite the fact that prisoners are highlighted as an at-risk group in the country.	descriptive, Required
	148	test_screen_prison_2_mx_q<br><br>Show the field ONLY if:<br>[contact_info_country] = '4' or [contact_info_country] = '5' or [contact_info_country] = '7' or [contact_info_country] = '10' or [contact_info_country] = '13' or [contact_info_country] = '17' or [contact_info_country] = '24' or [contact_info_country] = '25'	On a scale of 1-5, please indicate how well you believe this is functioning in practice in prisons in your country.	radio (Matrix), Required<br>0 Not functioning at all in practice (1)<br>1 Slightly functioning in practice (2)<br>2 Moderately functioning in practice (3)<br>3 Mostly functioning in practice (4)<br>4 Fully functioning (5)
	149	test_screen_prison_3<br><br>Show the field ONLY if:<br>[contact_info_country] = '1' or [contact_info_country] = '2' or [contact_info_country] = '9' or [contact_info_country] = '11' or [contact_info_country] = '12' or [contact_info_country] = '15' or [contact_info_country] = '19' or [contact_info_country] = '20' or [contact_info_country] = '21' or [contact_info_country] = '23' or [contact_info_country] = '26' or [contact_info_country] = '29'	A recent review8 of the state of viral hepatitis care in European prisons found that your country provides testing and/or screening for HCV in prisons, but the extent of coverage is unknown and prisoners are not highlighted as an at-risk group in the country.	descriptive, Required

	150	test_screen_prison_3_mx_q<br><br>Show the field ONLY if: [contact_info_country] = '1' or [contact_info_country] = '2' or [contact_info_country] = '9' or [contact_info_country] = '11' or [contact_info_country] = '12' or [contact_info_country] = '15' or [contact_info_country] = '19' or [contact_info_country] = '20' or [contact_info_country] = '21' or [contact_info_country] = '23' or [contact_info_country] = '26' or [contact_info_country] = '29'	On a scale of 1-5, please indicate how well you believe this is functioning in practice in prisons in your country.	radio (Matrix), Required<br>0 Not functioning at all in practice (1)<br>1 Slightly functioning in practice (2)<br>2 Moderately functioning in practice (3)<br>3 Mostly functioning in practice (4)<br>4 Fully functioning (5)

	151	test_screen_prison_ci_mx<br><br>Show the field ONLY if: [contact_info_country] = '30' or [contact_info_country] = '4' or [contact_info_country] = '5' or [contact_info_country] = '7' or [contact_info_country] = '10' or [contact_info_country] = '13' or [contact_info_country] = '17' or [contact_info_country] = '24' or [contact_info_country] = '25' or [contact_info_country] = '1' or [contact_info_country] = '2' or [contact_info_country] = '9' or [contact_info_country] = '11' or [contact_info_country] = '12' or [contact_info_country] = '15' or [contact_info_country] = '19' or [contact_info_country] = '20' or [contact_info_country] = '21' or [contact_info_country] = '23' or [contact_info_country] = '26' or [contact_info_country] = '29' or [contact_info_country] = '1' or [contact_info_country] = '2' or [contact_info_country] = '9' or [contact_info_country] = '11' or [contact_info_country] = '12' or [contact_info_country] = '15' or [contact_info_country] = '19' or [contact_info_country] = '20' or [contact_info_country] = '21' or [contact_info_country] = '23' or [contact_info_country] = '26' or [contact_info_country] = '29'	How informed do you consider yourself to be on the subject of this question?	radio (Matrix), Required<br>0 Not informed<br>3 Slightly informed<br>2 Mostly informed<br>1 Very informed

	152	test_screen_prison_com<br><br>Show the field ONLY if: [contact_info_country] = '30' or [contact_info_country] = '4' or [contact_info_country] = '5' or [contact_info_country] = '7' or [contact_info_country] = '10' or [contact_info_country] = '13' or [contact_info_country] = '17' or [contact_info_country] = '24' or [contact_info_country] = '25' or [contact_info_country] = '1' or [contact_info_country] = '2' or [contact_info_country] = '9' or [contact_info_country] = '11' or [contact_info_country] = '12' or [contact_info_country] = '15' or [contact_info_country] = '19' or [contact_info_country] = '20' or [contact_info_country] = '21' or [contact_info_country] = '23' or [contact_info_country] = '26' or [contact_info_country] = '29' or [contact_info_country] = '1' or [contact_info_country] = '2' or [contact_info_country] = '9' or [contact_info_country] = '11' or [contact_info_country] = '12' or [contact_info_country] = '15' or [contact_info_country] = '19' or [contact_info_country] = '20' or [contact_info_country] = '21' or [contact_info_country] = '23' or [contact_info_country] = '26' or [contact_info_country] = '29'	Additional comments:	notes

	153	treat_prison<br><br>Show the field ONLY if: [contact_info_country] = '1' or [contact_info_country] = '2' or [contact_info_country] = '4' or [contact_info_country] = '7' or [contact_info_country] = '9' or [contact_info_country] = '10' or [contact_info_country] = '11' or [contact_info_country] = '12' or [contact_info_country] = '13' or [contact_info_country] = '15' or [contact_info_country] = '17' or [contact_info_country] = '20' or [contact_info_country] = '21' or [contact_info_country] = '22' or [contact_info_country] = '23' or [contact_info_country] = '24' or [contact_info_country] = '25' or [contact_info_country] = '26' or [contact_info_country] = '28' or [contact_info_country] = '29' or [contact_info_country] = '30'	According to a recent review8 of the state of viral hepatitis care in European prisons, your country provides HCV treatment in prisons.	descriptive<br><br>1 | General population 2, High-risk population(s) (please specify below) 0, None of the above 99, Do not know

	154	treat_prison_mx_1<br><br>Show the field ONLY if: [contact_info_country] = '1' or [contact_info_country] = '2' or [contact_info_country] = '4' or [contact_info_country] = '7' or [contact_info_country] = '9' or [contact_info_country] = '10' or [contact_info_country] = '11' or [contact_info_country] = '12' or [contact_info_country] = '13' or [contact_info_country] = '15' or [contact_info_country] = '17' or [contact_info_country] = '20' or [contact_info_country] = '21' or [contact_info_country] = '22' or [contact_info_country] = '23' or [contact_info_country] = '24' or [contact_info_country] = '25' or [contact_info_country] = '26' or [contact_info_country] = '28' or [contact_info_country] = '29' or [contact_info_country] = '30'	On a scale of 1-5, please indicate how well you believe this treatment provision is functioning in practice in prisons in your country.	radio (Matrix), Required<br>0 Not functioning at all in practice (1)<br>1 Slightly functioning in practice (2)<br>2 Moderately functioning in practice (3)<br>3 Mostly functioning in practice (4)<br>4 Fully functioning (5)
	155	treat_prison_ci_mx<br><br>Show the field ONLY if: [treat_prison_mx_1] = '0' or [treat_prison_mx_1] = '1' or [treat_prison_mx_1] = '2' or [treat_prison_mx_1] = '3' or [treat_prison_mx_1] = '4'	How informed do you consider yourself to be on the subject of this question?	radio (Matrix), Required<br>0 Not informed<br>3 Slightly informed<br>2 Mostly informed<br>1 Very informed

	156	treat_prison_com<br><br>Show the field ONLY if: [contact_info_country] = '1' or [contact_info_country] = '2' or [contact_info_country] = '4' or [contact_info_country] = '7' or [contact_info_country] = '9' or [contact_info_country] = '10' or [contact_info_country] = '11' or [contact_info_country] = '12' or [contact_info_country] = '13' or [contact_info_country] = '15' or [contact_info_country] = '17' or [contact_info_country] = '20' or [contact_info_country] = '21' or [contact_info_country] = '22' or [contact_info_country] = '23' or [contact_info_country] = '24' or [contact_info_country] = '25' or [contact_info_country] = '26' or [contact_info_country] = '28' or [contact_info_country] = '29' or [contact_info_country] = '30'	Additional comments:	notes
	157	end_warning	NOTICEONLY click on the "Submit" button below if you have completed your work on the survey.<br><br>If instead you wish to save your work and continue responding to the survey at a later time, please click on the "Save & Return Later" button. Make sure to write down your return code.	descriptive
	158	hepcore_2018_phase_ii_complete	Section Header: *Form Status*<br><br>Complete?	dropdown<br>0 Incomplete<br>1 Unverified<br>2 Complete
