# Supplementary material for: The Hep-CORE policy score: A European hepatitis C national policy implementation ranking based on patient organization data
Source: PLoS One. 2020 Jul 28;15(7):e0235715. doi: 10.1371/journal.pone.0235715 (PMC7386634; doi:10.1371/journal.pone.0235715)
Supplement: S2 File — This is the complete survey that was used to ask experts what should be included into the index. (PDF) [file pone.0235715.s004.pdf]

Hep-CORE variables selection version 2

Introduction Hep-CORE variable selection

Dear Hep-CORE study group member, thank you for your time to complete this short survey. Below, there are 2 questions about whether or not micro-elimination efforts and service integration initiatives represent a real commitment by governments towards the 2030 WHO HCV elimination goals in a European context. The results from the Hep-CORE study group members will be used to help us build a model to create an index on HCV policy implementation in Europe.

1. Does an HCV micro-elimination effort in the following groups represent a *real* commitment from a government towards the 2030 WHO HCV elimination goals? Remember to consider the wider European context and not just the reality in your own country.

	No commitment	Weak commitment	Neutral commitment	Moderate commitment	Strong commitment
Generational cohorts	○	○	○	○	○
Haemodialysis patients	○	○	○	○	○
Haemophilia patients	○	○	○	○	○
Men who have sex with men	○	○	○	○	○
Migrants from countries with high prevalence of HCV	○	○	○	○	○
Patients with advanced liver disease	○	○	○	○	○
People living with HIV	○	○	○	○	○
People who inject drugs	○	○	○	○	○
Prisoners	○	○	○	○	○
Sex workers	○	○	○	○	○
Thalassemia patients	○	○	○	○	○
Transgender people	○	○	○	○	○
Veteran/Military personnel	○	○	○	○	○

2. Does the integration of HCV testing and treatment with the following services represent a *real* commitment from a government towards the 2030 WHO HCV elimination goals? Remember to consider the wider European context and not just the reality in your own country.

	No commitment	Weak commitment	Neutral commitment	Moderate commitment	Strong commitment
Alcohol use services	○	○	○	○	○
Blood safety	○	○	○	○	○
Cancer prevention and management	○	○	○	○	○
Haemodialysis centers	○	○	○	○	○
Harm reduction services	○	○	○	○	○
HIV treatment clinics	○	○	○	○	○
Migrant health services	○	○	○	○	○
Non-communicable disease prevention and management	○	○	○	○	○
Sex and reproductive health including (STI clinics)	○	○	○	○	○
